# A Comprehensive Contamination Investigation of Bohai Bay Seawater: Antibiotics Occurrence, Distribution, Ecological Risks and Their Interactive Factors

**DOI:** 10.3390/ijerph20021599

**Published:** 2023-01-16

**Authors:** Liang Tian, Xiaofu Xu, Zulin Zhang, Yongzhen Ding, Keqiang Zhang, Suli Zhi

**Affiliations:** 1 School of Energy and Environmental Engineering, Hebei University of Technology, Tianjin 300132, China; 2 Agro-Environmental Protection Institute, Ministry of Agriculture and Rural Affairs, Tianjin 300191, China; 3 Tianjin Fisheries Research Institute, Tianjin 300457, China; 4 The James Hutton Institute, Craigiebuckler, Aberdeen AB15 8QH, UK; 5 Key Laboratory of Low-Carbon Green Agriculture in North China, Ministry of Agriculture and Rural Affairs, Beijing 100193, China

**Keywords:** antibiotics, trace elements, inter-relation, ecological risks, seawater

## Abstract

A comprehensive, large-scale coastal investigation of antibiotics in seawater from Bohai Bay is lacking. Therefore, in this study, we investigated the occurrence and ecological risks of 45 antibiotics belonging to 5 classes in seawater from Bohai Bay, as well as their inter-relation with trace elements and other contaminants. The results show that tetracyclines (TCs) were detected in the highest concentration among the five classes (in the range of 0.6–2.0 μg/L). The total concentrations of the five classes of antibiotics were detected in the following order: tetracyclines (TCs) > quinolones (QAs) > sulfonamides (SAs) > macrolides (MAs) > lactams (LAs). Higher antibiotic concentrations were detected at the sampling sites closest to the coast or the shipping port. Among seven trace elements, four were quantitatively detected, with Zn representing the highest concentration. Antibiotic residuals were found to be positively correlated with total organic carbon (TOC), conductivity (Ec) and suspended solids (SS); pH and NH_4_^+^-N usually showed a negative correlation with antibiotics; TN and TP also exhibited relationships with antibiotics. The risk quotient (RQ) was calculated for different antibiotics at different sites. It was found that antibiotics pose higher risks to algae than to invertebrates or fish; sulfamethoxazole, enrofloxacin and ofloxacin were all found to pose high risk to algae at some of the sampling sites. Structural equation model (SEM) results show that trace elements, antibiotic levels and EC50 are the main factors affecting the ecological risks of antibiotics.

## 1. Introduction

Since their advent, antibiotics have played a significant role in preventing or treating human and animal bacterial infections [1], as well as in growth promoting for livestock and aquaculture [2]. China has been reported as the largest producer and consumer of antibiotics in the world [3,4]; approximately 162,000 t of antibiotics were used in China in 2015 according to a report, among which 48% were used for human and 52% for animal breeding [5]. However, after use, many of these antibiotics cannot be completely absorbed or metabolized by animals’ bodies. Therefore, a considerable fraction (30–90%) is discharged into environmental media [6]. Although some antibiotics can be reduced to a certain degree by specific processes, most are pseudo-persistent in various environment media [7,8]. Moreover, the presence of antibiotics can induce the development and contribute to the dissemination of ARGs (antibiotic resistance genes) on a global scale [9,10]. This phenomenon is considered a global public health crisis by the WHO (World Health Organization) [11].

Antibiotic residues are currently ubiquitous in various environmental media, such as manure [3], water [12], sewage sludge [6], sediments [13], soils [14] and even foods [15]. An increasing number of studies have been reported with a focus on the high prevalence of antibiotics in these environmental media. However, very limited information is available with respect to the occurrence of antibiotics, in marine systems (such as gulfs, bays or seas) [15]. Marine systems are major sources of marine products and other supplies for humans, but they are also considered very important pollutant pools for many pollutants transferring to open seas [16]. The pollution of the seawater is closely related to people’s health. However, seawater monitoring is difficult, owing to the long distances that must be traveled and the high expense. Therefore, it is of great significance to provide information about the pollution status of marine systems.

Bohai Bay, located in the northern China, is the largest semi-enclosed bay in China. The Bohai coastal region is one of the three most important economic zones in China [17]; this region not only accounts for approximately 17% of the total population but also contributes approximately 25% of the total GDP of China [18]. However, rapid economic development, increasing human activity and hundreds of drains along the coast discharging into seawater have resulted in serious environmental pollution in the Bohai region [19,20]. The widespread occurrence of pollutants in Bohai Bay may result in food and drinking water pollution in China and even spread to other seas. However, the co-occurrence of antibiotic residues and other contaminants in the Bohai Bay area has been very limited to date.

Previous studies related to antibiotics in the seawater of Bohai Bay have mainly focused on three aspects. (1) Sporadic studies have reported on the presence of antibiotics in the seawater of Bohai Bay. Zhang et al. [21] reported on the occurrence and distribution of nine antibiotics in the Bohai Sea and the Yellow Sea of China. Zou et al. [22] investigated the occurrence of 21 antibiotics in the seawater of Bohai Bay. (2) Several studies have reported on the occurrence of antibiotics in sediments or animals in Bohai Sea. For example, Liu et al. [13] studied 17 antibiotics in sediments from the Bohai Sea, China. Zhang et al. [23] investigated the risk of 12 commonly detected pharmaceuticals and personal care products (PPCPs) in freshwater organisms of the Bohai Rim, China. (3) Some studies have investigated other indicators in seawater from Bohai Bay. Li et al. [12] investigated and assessed the trace metal contents in coastal sediments from the Bohai Sea. Ding et al. [24] presented a report on the distribution and assessment of heavy metal pollution in surface sediments in Bohai Bay, China. Du et al. [1] reported the relationship between dissolved organic carbon (DOC), salinity, distance and the total concentration of antibiotics. However, these existing studies are subject to some limitations. First, comprehensive, large-scale coastal investigations on antibiotics in the seawater from Bohai Bay are lacking (additional valuable information should be provided in a timely manner to supplement existing reports). Secondly, the antibiotic species investigated in existing reports are limited (≤21); for example, antibiotics used in both humans and animals (i.e., macrolides, lactams, etc.) are rarely reported. Thirdly, information on the relationship between different contaminants in seawater from Bohai Bay has been scarcely reported, especially the relationship between trace elements and antibiotics.

Therefore, in this study, we conducted a comprehensive contamination investigation of Bohai Bay seawater, focusing on the occurrence, distribution, inter-relation, and ecological risks of a rich variety of antibiotics (45 kinds). Moreover, trace elements and environmental factors were also comprehensively investigated in order to analyze their correlation with antibiotic residues. In total, 45 widely used antibiotics (5 groups: TCs, SAs, QNs, MLs and LAs) and 7 representative and common trace elements (Pb, Cd, Cu, Cr, Zn, As and Hg) were investigated, as well as electric conductivity (Ec), suspended solids (SS), pH value, total organic carbon (TOC), total phosphorus (TP), total nitrogen (TN) and ammonia nitrogen (NH_4_^+^-N) levels. The aims of this study were (1) to analyze the occurrence and distribution of antibiotics in seawater of Bohai Bay, (2) to investigate trace elements and environmental factors that may interact with antibiotics and (3) to evaluate the ecological risks posed by antibiotics for aquatic animals and plants in Bohai Sea.

## 2. Materials and Methods

### 2.1. Materials

Methanol (MeOH), acetonitrile (ACN) and formic acid of HPLC grade were purchased from Thermo Fisher Scientific (USA). Na_2_-EDTA (disodium ethylene diamine tetra-acetic acid) was purchased from Sinopharm Group Co., Ltd. (Tianjin, China). All standards of antibiotics were obtained from Dr. Ehrenstorfer Gmbh (Augsburg, Germany). The full names and abbreviations of the 45 selected antibiotics are shown in Table 1. Standard 100 ppm stock solutions were prepared in MeOH or ACN and stored at −20 °C away from light. Working standard solutions were obtained by diluting the abovementioned stock solutions to specific concentrations.

### 2.2. Sample Preparation

#### 2.2.1. Sample Collection

**Figure 1:**
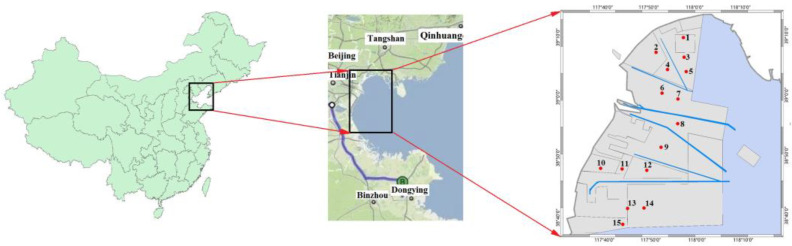
The locations of Bohai Sea and sampling sites.

Samples were collected around the Bohai Sea, which covers an area of 2146 km^2^. Water samples were collected in May (dry season) 2018 during cruises on a research vessel. The sample locations are shown in Figure 1. The sample locations were set around the shipping lane. All the samples were obtained from the Bohai Sea using a designed sampler under approximately 10–50 cm under the water surface. Three parallel samples were collected at each sampling site. All water samples were transferred to sample bottles immediately, which were precleaned thoroughly before sampling. Before the cruise ship returned, all samples were stored at 4 °C in a refrigerator in the dark. Then, they were further treated and analyzed in the laboratory.

#### 2.2.2. Sample Preparation

The sample volume was 400 mL. In order to reduce the binding between antibiotics and cations, 0.2 g Na_2_EDTA•2H_2_O was added to each sample before extraction. The pH values of the samples were adjusted to approximately 3.0 by 50% formic acid (*v*:*v* = 1:1). Then, the samples were simply filtered and centrifuged at 4000 rpm for 20 min. Solid-phase extraction (SPE) was performed with a PRiME HLB cartridge to purify the supernatant at a flow rate of approximately 10 mL/min. Then, 6 mL ultrapure water with 5% MeOH was used to wash the PRiME HLB cartridges, followed by vacuum drying for 20 min. Next, 6 mL MeOH and ACN (*v*:*v* = 3:7) containing 5‰ formic acid was used to elute the SPE cartridges. The eluents were dried by nitrogen using an N-EVAP 112 nitrogen evaporator (Oganomation Associates Inc., www.organomation.com, Norcross, GA, USA) at a temperature of 40 °C. Then, the dry matter was dissolved in 1 mL of MeOH:water = 1:1 (*v*:*v*) organic solvent containing 5‰ formic acid. The final mixtures were filtered by nylon syringe filters with 0.22 μm pore diameter and stored at −20 °C before analysis.

#### 2.2.3. Sample Analysis

The target antibiotics were analyzed by high-performance liquid chromatography–tandem mass spectrometry. An ACQUITY UPLCAB SCIEX QTRAP^®^4500 (Waters, Milford, MA, USA) equipped with an ACQUITY UPLC^®^HSS T3 C18 COLUMN (2.1 mm × 100 mm, 1.8 μm) was used to test the antibiotic concentrations. Exactly 2 μL of sample was injected to quantify the concentrations of antibiotics. The flow rate was selected as 0.3 mL/min. ACN was selected as mobile phase A, and 0.1% aqueous formic acid was chosen as mobile phase B. The following program of gradient elution was conducted: for 0–1.5 min, phases A and B were 10% and 90%, respectively; within 5.5 min, phase A was ascended to 80%; the ratios of phases A and B were held for 2 min; then, phase A was increased to 90%, and phase B was decreased to 10% for 7.5 min; then, for 12 min, phase A was ramped to 10%. Antibiotic concentrations were calculated according to matrix-matched standard curves. The MS/MS parameters were optimized using the methods described by Zhi et al. [3].

### 2.3. Environmental Factors and Trace Element Analysis

pH and electrical conductivity (Ec) were determined by the electrode test method. Suspended solids (SS) were determined by the weighing method after drying. Total organic carbon (TOC) was determined by the oxidation method. TP, TN and NH_4_^+^-N were measured by spectrophotometry. The concentrations of trace elements (Zn, Cu, Zn, Pb, Cr, Cd As and Hg) were analyzed by inductively coupled plasma mass spectrometry (ICP-MS) according to the procedure described in a previous study [19].

### 2.4. Risk Assessment

The ecological risk of antibiotics can be assessed by the risk quotient (RQ) method, which is widely used to assess various environmental pollutants. The RQ of antibiotics in the aquatic environment can be calculated according to the following equation:

RQ = MEC/PNEC(1)

where MEC is the measured environmental concentration, and PNEC is the predicted no-effect concentration in water. The PNEC in water medium was calculated according to the following equation:

PNEC = (LC_50_ or EC_50_)/AF (2)

where LC_50_ or EC_50_ represents the lowest median effective concentration value, which can be obtained from the reported data [25,26,27,28,29,30]. As reported, AF represents an appropriate standard assessment factor (1000).

## 3. Results and Discussion

### 3.1. Overall Residual Levels of Different Antibiotic Classes

**Figure 2:**
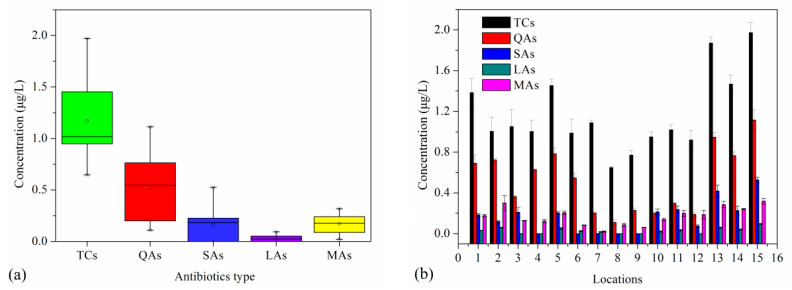
Total antibiotic concentrations (**a**) and distributions at different sampling sites (**b**) in seawater.

In this study, we investigated five classes of antibiotics: TCs, QAs, SAs, LAs and MAs. Figure 2 represents the total concentrations of different classes of antibiotics in Bohai seawater. All classes of antibiotics were quantitatively detected in the samples. As shown in Figure 2a, TCs represented the highest concentration among the five classes (in the range of 0.6–2.0 μg/L), whereas LAs had the lowest concentration (in the range of ND-0.1 μg/L). It was previously reported that TCs are widely used in aquaculture because they are broad-spectrum, inexpensive drugs [3]. Therefore, TCs are usually detected with high residual levels in environmental media. The overall concentrations of different classes of antibiotics in the seawater of Bohai Bay have not been frequently reported. Li et al. [15] reported on the total concentrations of some antibiotics in mollusks from Bohai Sea (not seawater) and showed that the concentration of QAs in mollusks ranged from 8.79 to 557.00 mg/kg. Moreover, researchers have reported results on antibiotics concentrations in several rivers and lakes in China. For example, Li et al. [31] studied antibiotics in Baiyangdian Lake in northern China and showed that SAs had the highest residual levels (in the range of 0.86 to 1562 ng/L; mean value of 383 ng/L). These values are higher than those detected in seawater from Bohai Bay in the present study. Figure 2b shows the total residual concentrations of antibiotics at different sampling sites. Relatively higher antibiotic concentrations were detected at sampling sites 1, 2 and 10, which may be due to the relatively shorter distance to beach from these sites relative to other sampling sites. These sites might receive more pollution from human activities, making seawater quality more susceptible. Relatively high antibiotic concentrations were also detected at sampling sites 13–15, which may be due to the port activities that occur near these sites. Sampling sites far from beaches (e.g., sites 7, 8, 9 and 12) were usually found to have relatively low antibiotic concentrations. Antibiotics in seawater usually originate from river water from the mainland; therefore, water at the mouth of rivers coming into contact with seawater was found to have relatively high antibiotic concentrations. Similar results were obtained in previous studies. Zou et al. [22] showed that human activities have some effects on the residual concentrations of antibiotics in the environment. However, these antibiotics may be gradually attenuated during the transport process into deep-sea areas as a result of physicochemical processes such as adsorption, dilution, photolysis, hydrolysis and/or some biological processes (biodegradation, etc.) [21].

### 3.2. Residual Levels of Single Antibiotic Concentration

#### 3.2.1. TCs Levels

**Figure 3:**
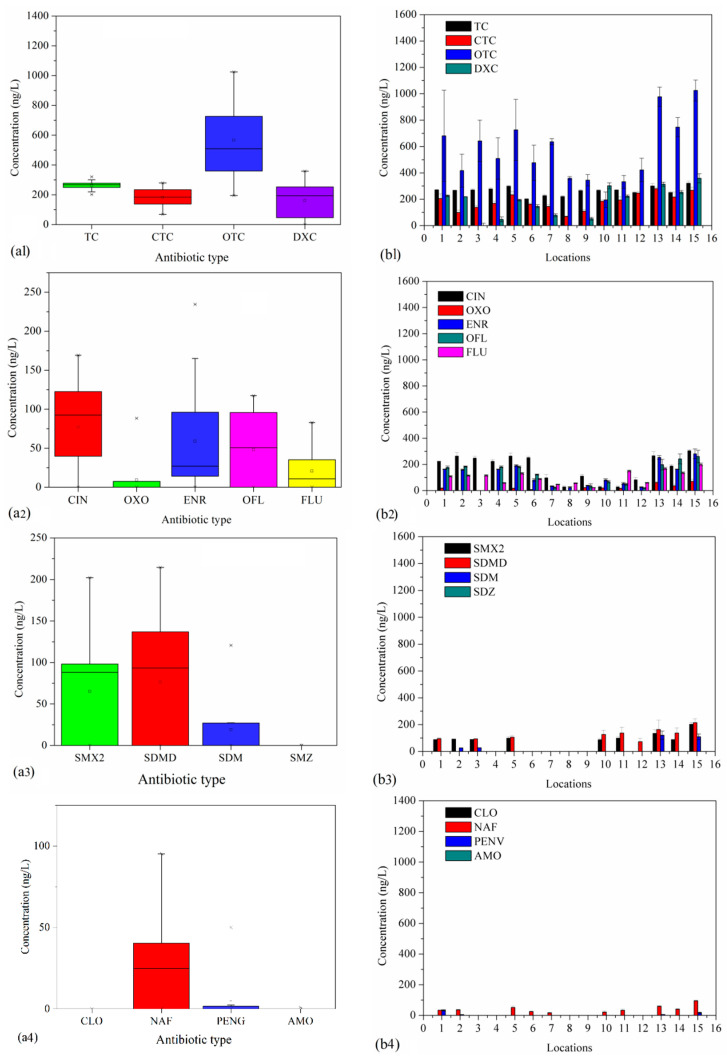

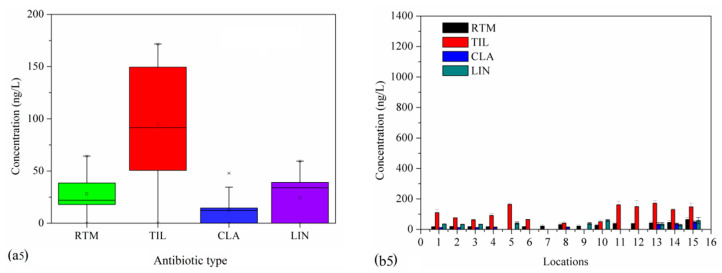
Single antibiotic concentrations ((**a1**–**a5**) for TCs, QAs. SAs, LAs, and MAs, respectively) and their distributions at different sampling sites ((**b1**–**b5**) for TCs, QAs. SAs, LAs, and MAs, respectively) in seawater.

Figure 3a1,b1 show the single concentration of TCs in seawater from Bohai Bay. Figure 3a1 shows the overall profile of different types of antibiotics. It shows that TCs, CTCs, OTCs and DXCs were quantitatively detected in the seawater samples in the range of 201.7–321.2 ng/L, 68.7–279.9 ng/L, 195.0–1024.7 ng/L and ND-358.5 ng/L, respectively. The mean values of TC concentration in the seawater samples were detected in the order of OTC > CTC > TC > DXC. Zhang et al. [32] also reported that OTCs were detected in the highest concentration (200.9 ± 22.8 ng/L) in seawater in an investigation conducted in Bohai Bay. Liu et al. [13] reported a range of 2.2–4695 μg/kg for OTCs in surface sediments around Bohai Sea. The possible reason for such high concentrations could be that OTCs are the main antibiotics used in mariculture [32]. Yang et al. [33] published a review on antibiotics in different lakes and showed that TCs represent an important class in lakes and rivers. Chen and Zhou [34] reported that the concentrations of OTCs in Huangpujiang River ranged from “not detected” to 219.8 ng/L. Wang et al. [7] reported an order of TC (745.2 ng/L) > OTC (682.9 ng/L) > CTC (426.0 ng/L) according to their detection in Honghu Lake. All these results are similar to those obtained in the present study.

Figure 3b1 shows the profiles of TCs at different sampling sites. OTCs and CTCs show a similar trend at the sampling sites, with higher residual concentrations at sites 1–3, 10 and 13–15, all of which are close to the coast. Xie et al. [26] reported a negative relationship between the organic pollutant concentration and the distance of sample sites from the coast in the Bohai Sea. This may be because sites close to shorelines are more susceptible to pollution. However, TCs and DXCs presented different profiles at sampling sites, although all types were detected at high concentrations at site 15. This may be due to the port activities occurring near site 15.

#### 3.2.2. QA Levels

QAs were also detected in high concentrations in seawater from Bohai Bay. Figure 3a2,b2 show the single concentrations of QAs in seawater from Bohai Bay. As shown in Figure 3a2, five types of QAs were quantitatively detected: CINs, OXOs, ENRs, OFLs and FLUs. Their concentrations were ND-169.2 ng/L, ND-23.1 ng/L, ND-234.4 ng/L, ND-117.3 ng/L and ND-83.0 ng/L, respectively. The mean concentrations of QAs in the seawater were detected in the order of “CIN (77.2 ng/L) > ENR (58.9 ng/L) > OFL (47.9 ng/L) > FLU (20.7 ng/L) > OXO (9.3 ng/L)”. Zou et al. [22] investigated the variation of antibiotic concentrations in Bohai Bay and reported concentration ranges for OFL, NOR and CIP of ND-5100 ng/L, ND-6800 ng/L and ND-390 ng/L, respectively. Li et al. [35] studied antibiotics in Xiaoqing River, which is along the urbanizing Bohai Rim, and reported that the concentrations of OFL and NOR were 122.7 ng/L and 39 ng/L, respectively. These values are much higher than the results obtained in this study. However, Zhang et al. [32] reported that just one quinolone (ENR) was detected in the seawater of Bohai Bay. These conflicting results may be due to the different sampling sites, different sampling dates and different physicochemical parameters of the samples. Figure 3b2 shows that seawater at sites 1, 2, 4 and 13–15 had higher antibiotic concentrations than seawater from other sites. A possible reason is that sites close to the coastline or the port are more affected by antibiotics. When antibiotics are discharged into the seawater, they can be attenuated during various processes. On one hand, they could be physically diluted as they are transported by ocean currents or by molecular thermal diffusion. On the other hand, antibiotics can be easily adsorbed by suspended particles or sediments, resulting in concentration reduction. In addition, some physiochemical processes (e.g., hydrolysis and photolysis) or biological processes (biodegradation) result in reductions in the concentrations of antibiotics. The above reasons all contribute to decreases in the concentrations of antibiotics. Similar results were reported by Zhang et al. [21].

#### 3.2.3. SAs Levels

As shown in Figure 3a3,b3, only three types of SAs were quantitatively detected in seawater from Bohai Bay. The concentrations of SMX2, SDMD and SDM were ND-202.3 ng/L, ND-214.4 ng/L and ND-120.7 ng/L, respectively, with mean values in the order of SMX2 (65.1 ng/L) > SDMD (62.8 ng/L) > SDM (19.0 ng/L). Niu et al. [20] showed that SMX2 had the highest concentration of 1454.2 ng/L in Bohai seawater, which is much higher than the concentrations detected in our study. Zou et al. [22] also reported high concentrations of SAs of ND-140 ng/L, ND-41 ng/L and ND-130 ng/L for SMX2, SDZ and SDM, respectively. However, Zhang et al. [21] reported that SMX2, SDZ and SDM had concentration ranges of ND-8.3 ng/L, ND-0.36 ng/L and ND-0.16 ng/L, respectively, are lower than those obtained in the present study. These conflicting results may be due to the different sampling sites, sampling dates and physicochemical parameters of the samples. Some researchers have reported that SAs were gradually replaced by other classes of antibiotics, such as lactams or macrolides, but that they can also be detected as a result of use in poultry and aquaculture, owing to their low price [22]. In addition, some reports have shown that SAs are commonly used in human medicine [8,36]. Therefore, SAs in seawater may partially originate from aquaculture zones and domestic wastewater. Moreover, no antibiotics were detected at sites 4 and 6–8, which is in accord with the above conclusion, as these sites are relatively far from the coastline.

#### 3.2.4. LA and MA Levels

LAs and MAs are two classes of antibiotics that have been increasingly used in recent years, gradually replacing some types of TCs and SAs both for human and animal use. Therefore, a comprehensive study of LAs and MAs is needed. A total of two types of LAs and four types of MAs were quantitatively detected (Figure 3a4,a5). The concentrations of LAs NAFs and PENGs were in the ranges of ND-95.2 ng/L (mean, 27.6 ng/L) and ND-50.0 ng/L (mean, 4.3 ng/L), respectively. The concentrations of MAs RTMs and TILs were in the ranges of ND-64.3 ng/L (mean, 28.2 ng/L) and ND-171.7 ng/L (mean, 95.0 ng/L), respectively. LAs have rarely been reported in seawater from Bohai Bay. For example, Niu et al. [20] and Zou et al. [22] studied the occurrence of antibiotics in seawater from Bohai Bay but reported no types of Las and only a few MAs. Niu et al. [20] reported ERY and RTM in the ranges of 0.6–38.2 ng/L and 0.6–17.8 ng/L, respectively, in seawater from Bohai Bay. Zou et al. [22] reported ERY and RTM in the ranges of ND-150 ng/L and ND-630 ng/L, respectively, in seawater from Bohai Bay. These values are much higher than those obtained in the present study. Figure 3b4,b5 show that the concentrations of LAs differed depending on the sampling site. Attenuation along the coastline was also observed for LAs and MAs.

### 3.3. Residual Levels of Trace Elements

China is considered a world leader in the field of manufacturing, but manufacturing activities have caused considerable pollution in the country [35]. Trace elements are one of the most prevalent classes of pollutants. Table 2 shows the trace element concentrations in seawater from Bohai Bay at different sites. Cu, Cr and Hg were not quantitatively detected. The concentrations for Zn were highest (in the range of 120.0 ± 8.2–320.0 ± 35.6 μg/L). The concentrations of Pb, Cd and As were 21.8 ± 0.3–62.8 ± 5.1 μg/L, 5.7 ± 0.4–11.4 ± 0.9 μg/L and 0.7 ± 0.1–5.8 ± 0.1 μg/L, respectively. The mean concentration of Zn was higher than the limit (50 μg/L) set by environmental quality standards for surface and fishery water (MEP, Environmental Quality Standards for Surface Water, 2002). Su et al. [37] reported that the As, Zn, Cu and Ni concentrations in Bohai Rim rivers were 0.01–347.7 μg/L, 0.035–25,370 μg/L, 0.0007–2755 μg/L and 0.8–571 μg/L, respectively. Liang et al. [19] reported that the mean concentrations of Zn, Cu, Cr, As, Pb and Cd were 49.0 μg/L, 43.8 μg/L, 33.9 μg/L, 11.3 μg/L, 3.42 μg/L and 0.21 μg/L, respectively, in the rivers draining into the Bohai Sea. Trace elements were detected at the highest concentrations at sampling sites 5, 6, 13 and 15. Trace elements are not easily degradable pollutants, so were not attenuated along the coastline.

### 3.4. Variation of Environmental Factors

**Figure 4:**
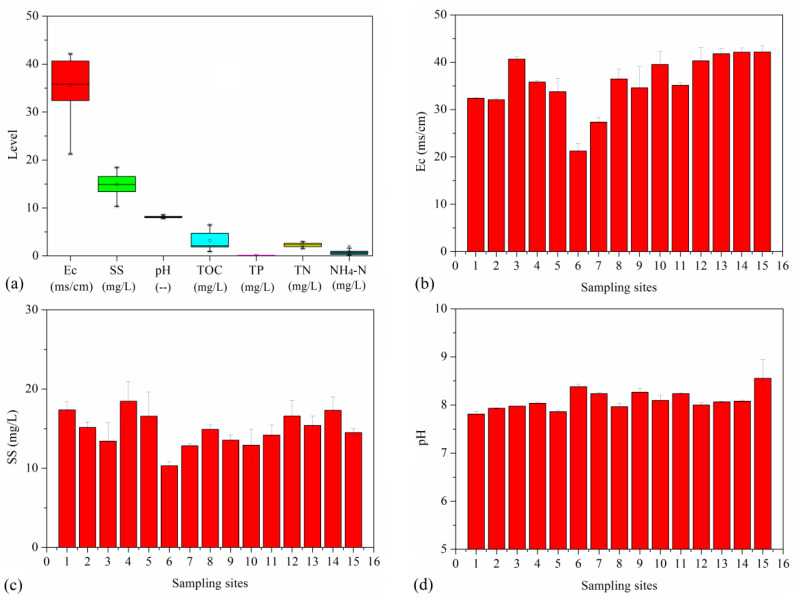

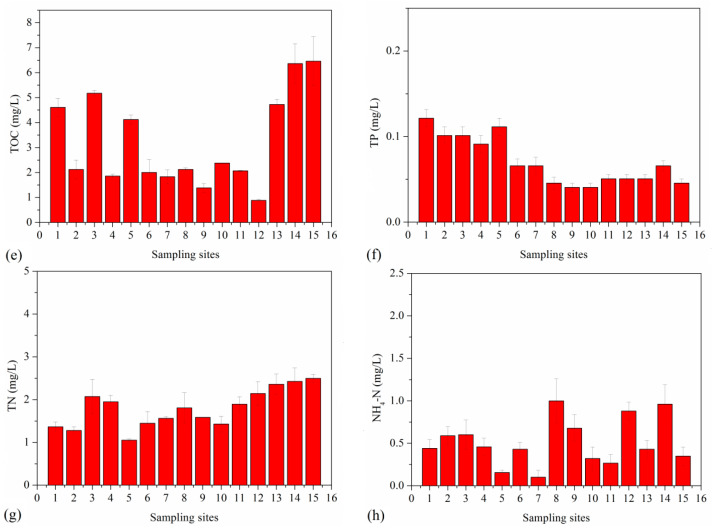
Environmental factor profiles at different sampling sites in seawater from Bohai Bay (**a**): total concentration range; (**b**): Ec of samples; (**c**): SS of samples; (**d**): pH of samples; (**e**): TOC of samples; (**f**): TP of samples; (**g**): TN of samples; (**h**): NH_4_^+^-N of samples.

In this study, we investigated the environmental factors affecting seawater from Bohai Bay. Figure 4a shows the total concentration profile of these environmental factors. Ec, SS, pH, TOC, TP, TN and NH_4_^+^-N were measured in the ranges of 21.2–42.1 ms/cm, 10.3–18.5 mg/L, 7.8–8.6, 1.4–6.5 mg/L, 0.0–0.1 mg/L, 1.1–2.5 mg/L and 0.1–1.0 mg/L, respectively. Seawater from sites 6 and 7 had relative low Ec values, which may due to the distance of these sites from the coastline (Figure 4b). Figure 4c,d shows that SS and pH values were similar at all the sampling sites. Figure 4e shows that obvious high TOC values were obtained at sites 1, 3, 5 and 13–15. Figure 4f,g show that TP and TN in the seawater presented different variation trends: high TP values were obtained at sites 1–5, whereas high TN values were obtained at sites 13–15. Figure 4h shows high NH_4_^+^-N contents were obtained at sampling sites 8, 12 and 14. The values for these environmental factors have been scarcely reported, and they represent distinct trends. Therefore, these environmental factors may have varying relationships with antibiotic concentrations (analyzed in Section 3.6).

### 3.5. Correlation with Antibiotics

**Figure 5:**
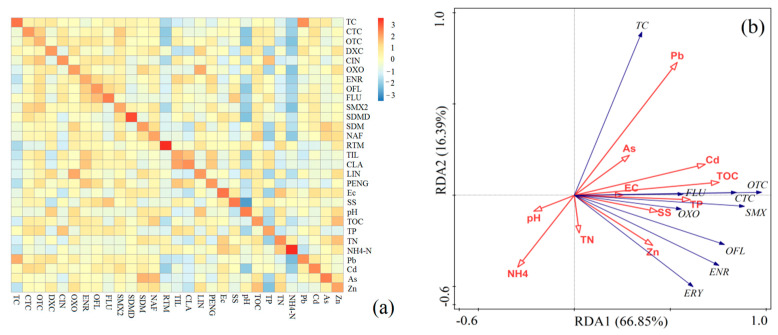
Correlations (**a**) and redundancy analysis (**b**) among antibiotics, trace elements and environmental factors.

The relationships among environmental parameters, trace elements and antibiotic concentrations were evaluated. Figure 5a shows that each antibiotic is obviously affected by TOC. Wenk et al. [38] found that TOC had a considerable effect on antibiotic transformation processes (such as photodegradation and photosensitization). Most antibiotics were found to have negative relationships with pH values and NH_4_^+^-N. Liu et al. [38] also reported that some antibiotics (e.g., NOR) can be negatively affected by pH values. A possible reason for this effect might be that antibiotics are much easier to dissolve and ionize in acidic environments. Ec and SS were found to have a positive relationship with antibiotics. TN and TP were also found to have some relationships with antibiotics. Liu et al. [39] showed that sulfadiazine was positively correlated with TN, TP and Ec and that trimethoprim was negatively correlated with TN, TP and Ec. Li et al. [12] showed that some environmental factors (COD_Mn_, NH_4_^+^-N and TN) can strongly affect the distribution of TCs, SAs and AGs in water. Trace elements usually have a positive relationship with antibiotics, although scarcely reported in existing studies. In addition, some trace elements were found to have relationships with environmental factors. For example, Zn presented a significantly positive relationship with TOC, and As was found to have positive relationships with TOC and TN. Figure 5b shows a redundancy analysis of antibiotics, trace elements and environmental factors. The results show that TOC explains 38.9% of the changes in antibiotics in the investigated water samples, in accordance with the fact that TOC was found to have a strong relationship with antibiotics (Figure 5a). Moreover, some trace elements explain a considerable amount of changes in antibiotic: Pb explains 15.5%, and Zn explains 9.8%. In addition, TP and TN explain 12.8% and 2.8% of antibiotic variation, respectively.

### 3.6. Ecological Risk

**Figure 6:**
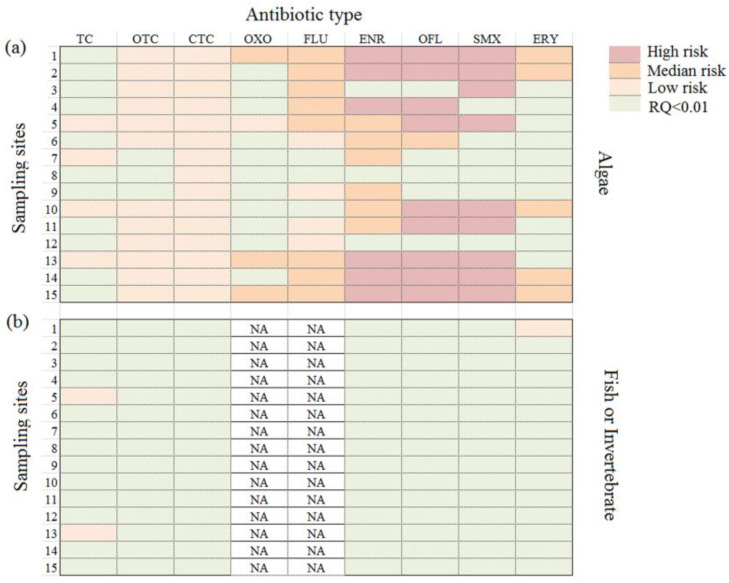
Risk quotients (RQs) of the detected antibiotics for algae (**a**) and fish or invertebrates (**b**).

The presence of antibiotics in seawater may pose toxicity risk for aquatic organisms. The risk quotient (RQ) method has been widely used to evaluate the ecological risks of antibiotics to aquatic organisms. Different trophic-level species exhibit different sensitivities to antibiotics [28]. Therefore, in this study, we investigated the RQs of the detected antibiotics for representative aquatic species (algae and invertebrates or fish), as shown in Figure 6. As reported, RQ > 1 indicates high risk, 0.1 < RQ < 1 indicates median risk and 0.1 < RQ < 0.01 indicates low risk [26]. Figure 6a shows that SMX, ENR and OFL all exhibited high risk to algae at some sampling sites. This result is in accordance with the results reported by Xu et al. [27], who showed that OFL posed a high risk for algae during an investigation of seawater in the Pearl River Delta. Park and Choi [25] reported that SMX exhibited much more toxic effects on algae than some other antibiotics and was identified as a potential high-risk antibiotic to animals or plants in aquatic environments. The reason may be that SMX can inhibit phagocytic activity even at very low concentrations [40]. Figure 6b shows that the RQs of the detected antibiotics were very low, which means that these antibiotics are not likely to have toxic effects on invertebrates or fish. As shown in Figure 6a,b, the risks of antibiotics to algae seem to be more serious than those to invertebrates or fish. The reason may be that that low-trophic-level species (e.g., cyanobacteria and algae) are more sensitivity to antibiotics than higher-trophic-level organisms (e.g., crustaceans and fish) [28]. Some previous studies reported similar results. Xie et al. [26] also reported that algae were the most sensitive biota to the target antibiotics. Lützhøft et al. [41] showed that invertebrates were not affected by antibiotics as much as algae. Kümmerer [42] reported that fish also are not likely to be affected by antibiotics in aquatic environments. Similar results were reported in recent literature [15,26]. However, considering the potential for long-term bioaccumulation in invertebrate or fish muscles, the risks of antibiotic pollution to aquatic animals and plants cannot be ignored.

### 3.7. Direct and Indirect Effects on Ecological Risk

**Figure 7:**
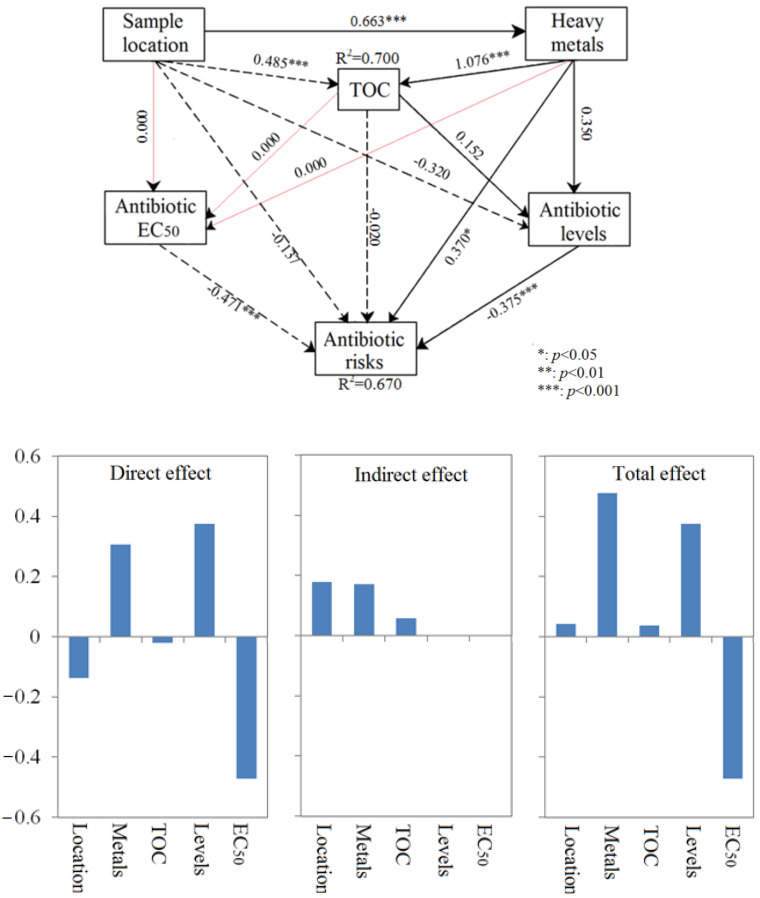
Structural equation models (SEMs) for the direct and indirect effects of sample location, trace elements, antibiotic levels, EC_50_ and TOC on ecological risks. Note: red line indicates no effect, black dotted line represents negative effect and the black solid line represents positive effect.

Structural equation models (SEMs) are predictive models used to calculate different effects of factors on target indicators. A prior approach and hypotheses were derived from possible inter-relationships between factors to construct an inter-relation network. SEMs could offer the capacity to visualize casual relationships and treat them as a system. It is useful to analyze the complex correlations in ecosystems [43]. SEMs can not only elucidate the direct effects of factors but also the indirect effects of factors. In the present study, the direct, indirect and total effects of sample location, trace elements, antibiotic levels, EC_50_ and TOC on ecological risks were determined by SEM, as shown in Figure 7. It is obvious that antibiotic levels have extremely significantly negative effects on ecological risks (*λ* = −0.471, *p* < 0.001), whereas EC_50_ has extremely significantly positive effects on ecological risks (*λ* = 0.375, *p* < 0.001). Both of these factors directly affect the antibiotic ecological risks (no indirect effects). This is easy to understand based on the way ecological risk is calculated in Equations (1) and (2). Trace elements also have significantly positive effects on ecological risks (*λ* = 0.370, *p* < 0.05) through direct and indirect positive effects. This suggests that there may be a link between trace elements and antibiotic pollution that needs to be further explored. It has been reported that antibiotics and metal ions can easily chelate together, making it difficult for antibiotics to dissociate [3]. Therefore, we should pay more attention to the ecological risk caused by complex pollution. Sample location was found to have obvious effects on TOC and trace elements, with no effect on EC_50_. Given the connection between these factors, we should pay more attention to the synergistic pollution effect between different compounds.

## 4. Conclusions

In this study, the variations of antibiotics, trace elements and other factors were investigated in seawater collected in Bohai Bay, China. TCs were found to be widely distributed in different samples, whereas relatively lower concentrations of MAs and LAs were detected. The highest concentration was 1024.7 ng/L for OTC in seawater. Zn was detected at higher concentrations than other elements. Different environmental factors were measured in the seawater, and their correlation coefficients with antibiotics were also analyzed. A risk assessment was conducted, the results of which reveal that some antibiotics present high ecological risks to algae but low risks to invertebrates and fish. Although this study provides some information, the combined toxicity of these pollutants needs further investigation.

## Figures and Tables

**Table 1:** Full names and abbreviations of antibiotics used in this study.

Full Name	Abbreviation	Full Name	Abbreviation
19 sulfonamide antibiotics (SAs)		13 quinolone antibiotics (QAs)	
sulfacetamide	SCM	ofloxacin	OFL
sulfisomidin	SIM	enrofloxacin	ENR
sulfadiazine	SDZ	difloxacin	DIF
sulfathiazole	STZ	danofloxacin	DAN
sulfamoxol	SMX1	nalidixic acid	NAL
sulfapyridine	SPD	lomefloxacin	LOM
sulfamerazine	SMR	sarafloxacin	SAR
sulfamonomethoxine	SMM	ciprofloxacin	CIP
sulfadimidine	SDMD	flumequine	FLU
sulfamethizole	SMZ	orbifloxacin	ORB
sulfadoxin	SDX	norfloxacin	NOR
sulfamethoxazole	SMX2	enoxacin	ENO
sulfisoxazole	SIX	oxolinic acid	OXO
sulfabenzamide	SB	6 macrolide antibiotics (MAs)	
sulfadimethoxine	SDM	roxithromycin	RTM
sulfaquinoxaline	SQX	clarithromycin	CLA
Sulfameter	SME	azithromycin	AZI
sulfaguanidine	SGN	spiramycin	SPI
sulfamethoxypyridazine	SMP	tilmicosin	TIL
5 tetracyclines (TCs)		Lincomycin	LIN
chlorotetracycline	CTC	2 lactam antibiotics (LAs)	
tetracycline	TC	penicillin G	PENG
oxytetracycline	OTC	oxacillin	OXA
doxycycline	DXC		
demeclocycline	DMC		

**Table 2:** Trace element contents in seawater from Bohai Bay (μg/L).

Site	Zn	Pb	Cd	As	Hg	Cu	Cr
1	174.1 ± 4.7	23.2 ± 0.0	8.6 ± 0.8	1.7 ± 0.1	-	-	-
2	163.3 ± 12.5	23.2 ± 0.0	9.7 ± 0.1	1.4 ± 0.1	-	-	-
3	161.5 ± 10.2	21.8 ± 0.3	10.9 ± 0.6	0.7 ± 0.1	-	-	-
4	166.7 ± 17.0	33.4 ± 1.0	10.0 ± 1.1	1.0 ± 0.0	-	-	-
5	120.0 ± 8.2	62.8 ± 5.1	10.2 ± 1.4	2.1 ± 0.0	-	-	-
6	176.7 ± 9.4	24.3 ± 0.1	6.2 ± 0.2	1.6 ± 0.0	-	-	-
7	133.4 ± 11.2	24.5 ± 0.6	5.7 ± 0.4	1.5 ± 0.0	-	-	-
8	140.0 ± 6.0	22.0 ± 1.0	6.0 ± 0.4	1.4 ± 0.0	-	-	-
9	156.7 ± 9.4	22.1 ± 2.8	7.2 ± 0.3	1.3 ± 0.0	-	-	-
10	153.3 ± 4.7	34.0 ± 2.9	9.9 ± 0.4	1.6 ± 0.0	-	-	-
11	180.0 ± 8.2	27.1 ± 0.9	8.2 ± 0.1	1.7 ± 0.0	-	-	-
12	162.6 ± 17.0	23.3 ± 0.9	7.5 ± 0.1	1.8 ± 0.0	-	-	-
13	226.5 ± 25.0	37.7 ± 5.7	11.4 ± 0.9	15.1 ± 0.1	-	-	-
14	286.9 ± 12.5	24.3 ± 0.9	7.9 ± 0.1	4.3 ± 0.2	-	-	-
15	320.0 ± 35.6	46.0 ± 0.1	9.8 ± 0.5	5.8 ± 0.1	-	-	-

## Data Availability

Data available on request for corresponding author.
